# Heat affected zone liquation cracking evaluation on FeMnAl alloys

**DOI:** 10.1080/14686996.2024.2342232

**Published:** 2024-04-19

**Authors:** Rafael Giorjao, Kaue C. Riffel, Eric Brizes, Katherine Sebeck, Antonio J. Ramirez

**Affiliations:** a Welding Engineering, Department of Materials Science and Engineering, The Ohio State University, Columbus, OH, USA; b Department of Aerospace Materials, NASA John H Glenn Research Center, Cleveland, OH, USA; c Department of Advanced Material Applications and Manufacturing, US Army Combat Capabilities Development Command Ground Vehicle Systems Center, Warren, MI, USA

**Keywords:** Spot varestraint testing, FeMnAl steels, CALPHAD, liquation cracking

## Abstract

FeMnAl steels are currently generating a lot of interest with potential applications for structural parts in armored vehicles due to their lower density and outstanding mechanical properties. Despite the extensive mechanical performance and heat treatment exploration of this alloy class, further weldability investigation is required for future large-scale deployment. In the present study, the liquation cracking of four heats of cast FeMnAl alloys was investigated by the spot-Varestraint technique. The study focuses primarily on the effect of the major elements of the FeMnAl system: C, Mn and Al. Optical and electron microscopy were employed to investigate the microstructural features, and CALPHAD was employed to aid the discussion regarding the alloy’s composition differences and their effect on the liquation cracking susceptibility. The study was able to identify that compositions with the higher Mn, C, and lower Al presented the highest liquation cracking susceptibility. Conversely, composition presenting lower Mn, C, and Al showed the most resistant behavior. Furthermore, lower Al content promoted a fully-γ microstructure at low temperatures, which encouraged the appearance of longer cracks as a γ-matrix is more susceptible to HAZ cracking than a fully ferritic (α) or duplex (α + γ) microstructure.

## IMPACT STATEMENT

The present manuscript indicates that FeMnAl steels are susceptible to liquation cracking for alloys with higher Mn, C, and lower Al.

## GRAPHICAL ABSTRACT

**Figure:**
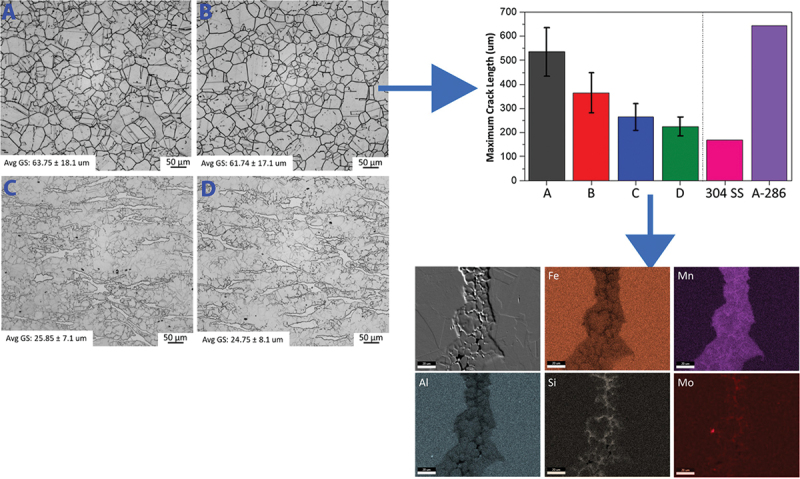

## 1. Introduction

Across US Army ground vehicle platforms, weight continues to increase in response to changing conditions on the battlefield. In some cases, the platforms are now exceeding the capabilities of support systems, affecting transportability or restricting the ability to incorporate additional upgrades. Considered a possible alternative to the current high-strength steels, FeMnAl steels take advantage of the density reduction promoted by additions of Al, while maintaining good mechanical properties such as high strength and high toughness.

FeMnAl (or Fe-Al-Mn-C) steels possess outstanding mechanical properties, with a yield strength of 0.4–1.0 GPa and ultimate tensile strength of 0.6–2.0 GPa [1–3]. The range of FeMnAl chemical compositions promotes different phases in equilibrium at room temperature, such as fully austenitic, fully ferritic, or duplex (ferrite and austenite) [2]. The combination of lower density, formability, age hardenability, and high energy absorption in a crash makes the FeMnAl alloys a potential candidate to address the growing demand for strengthening of vehicle reinforcing components [2,4–6]. Lightweight FeMnAl steel has been demonstrated to achieve a 1:1 ballistic efficiency and a 10–15% density reduction when cast and produced in laboratory-scale wrought plates [2].

Exploration of FeMnAl chemical composition has been heavily pursued to identify optimal mechanical performance. Studies focusing on single- or multi-element interactions established relationships with properties such as aging behavior and impact toughness [4,7–10]. Despite the extensive mechanical performance and heat treatment exploration of this alloy class [2,3,11–13], further weldability investigation is required for future large-scale deployment. Regarding cracking related to welding processes, two phenomena are the most impactful: solidification cracking that occurs in the fusion zone and heat-affected zone (HAZ) liquation cracking located in the partially melted zone. Solidification cracks are often identified by their fracture surfaces that reveal the dendritic morphology of the solidifying weld metal [14]. In contrast, liquation cracking occurs in the high-temperature HAZ adjacent to the fusion boundary and is associated with localized melting at grain boundaries due to the segregation of solidus-depressing elements. This type of cracking is often encountered during the welding of highly engineered and alloyed materials [14]. It is particularly prevalent in nickel- and aluminum-based alloys and fully austenitic stainless steels [15].

Metallurgically, HAZ liquation cracking is related to the presence of liquid films that are unable to accommodate the thermally and/or mechanically induced strain experienced during weld cooling. It is recognized that the simultaneous presence of a crack-susceptible microstructure and a critical level of restraint are necessary to promote cracking. Since the control of weld restraint is often difficult, a reduction in liquation cracking susceptibility is usually achieved by adjusting the composition and microstructure.

To date, over 150 separate and distinct techniques have been developed to quantify weld solidification and HAZ liquation cracking susceptibility. These tests vary widely in their approach and utility but can generally be classified as representative (self-restraint) or simulative (augmented restraint) test techniques. Among those, the longitudinal-Varestraint, spot-Varestraint, and hot-ductility tests are three of the few methods utilized to quantify the HAZ liquation cracking susceptibility and develop a fundamental understanding of the cracking phenomena.

Limited research has been conducted on joining FeMnAl plates, and minimal work has been performed on this system’s weldability. A preliminary investigation was conducted by Chou et al. [16,17], who studied the effect of carbon content on the weldability of Fe–30Mn–(8–10)Al–(0.12–1.17)C steels. According to the authors, a strong relationship between carbon content and ferrite fraction was confirmed, and the alloys with a significant fraction of ferrite exhibited an enhanced hot cracking resistance (solidification and liquation cracking). Kim et al. [18] confirmed via Varestraint testing that the precipitation of κ-carbides at high temperature exacerbates local HAZ liquation cracking. The experiments showed that intergranular cracking was a result of the relative weakness of the grain boundaries due to intergranular κ-carbide formation at high temperatures. Keil et al. [19], while performing Self-Restrained Testing on a Fe-17.8Mn-1.33Al-0.59C alloy, observed high susceptibility to liquation cracking irrespective of the filler material used. The author related the HAZ cracking performance of FeMnAl to the combination of a large solidification temperature range, poor heat conduction, high thermal expansion coefficients, and extreme grain growth in the HAZ.

The present study aims to support the selection of an optimal FeMnAl composition by evaluating the liquation cracking of four heats of cast FeMnAl alloys. The chemical compositions selected aimed to evaluate the effect of the major elements (C, Mn and Al) on the liquation cracking susceptibility of FeMnAl. The liquation cracking investigation was conducted using the spot-Varestraint test to evaluate, quantify, and compare the studied alloys’ susceptibility to this cracking phenomenon. Optical and electron microscopy were performed to investigate the microstructural features. Computer Coupling of Phase Diagrams (CALPHAD) analysis was employed to aid the discussion regarding the alloy’s composition differences and their effect on the liquation cracking susceptibility.

## 2. Materials and methods

Four different FeMnAl alloys were evaluated. The alloys were melted in an electric arc furnace and the bottom teemed with protective flux in order to eliminate unwanted elements such as S and P, which are known to cause severe weld cracking. The base plates were heat-treated at 950°C and subsequently quenched to room temperature. The four FeMnAl alloys were machined into specimens presenting the dimensions of 152.4 × 25.4 × 6.35 mm (6×1 × 0.25 in). The evaluated compositions followed additional efforts on investigating FeMnAl mechanical performance optimization through the covariance of the major alloying elements [4]. The materials were subjected to chemical composition analysis performed by an Optical Emission Spectrometer (OES) SPECTROMAXx Metal Analyzer, calibrated with high-Mn, and high-Al standards. The material composition taken from the cross-section of the plates is displayed below in Table 1.

**Table 1.** Alloy’s composition measured by optical emission spectroscopy in %wt.

Alloy	Fe	C	Mn	Al	Si	Mo	S	P
A	Bal	0.9	28.8	8.1	0.73	0.44	<0.0002	<0.005
B	Bal	0.8	28.9	8.2	0.72	0.45	<0.0002	<0.005
C	Bal	0.9	24.5	10.6	0.71	0.45	<0.0002	<0.005
D	Bal	0.8	24.4	10.4	0.72	0.45	<0.0002	<0.005

**Figure 1.**
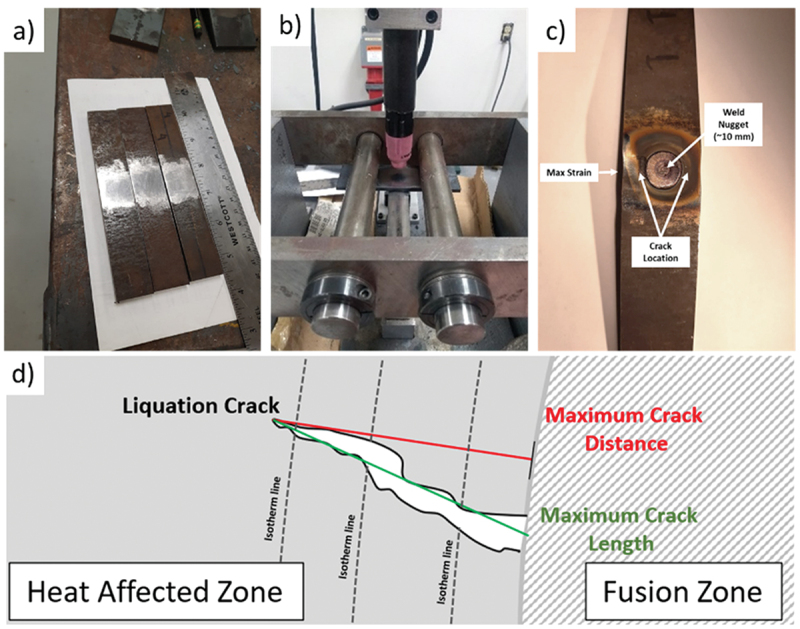
Spot Varestraint experiment set-up and tested sample features: (a) Samples geometry; (b) Spot Varestraint machine set-up; (c) Spot Varestraint samples crack location and max strain region; (d) Liquation crack schematic depicting the maximum crack length and maximum crack distance measurements.

As seen in Figure 1(a), samples were ground before the welding procedure to avoid contamination in the evaluated area. During spot-Varestraint testing, a Gas Tungsten Arc (GTA) spot weld is produced in the center section of the specimen, as seen in Figure 1(b). After a predetermined weld time, the arc is extinguished, and the specimen is forced to conform to the surface of a radiused die block. The augmented strain is adjusted by changing the die block radius. The parameters listed in Table 2 were based on a previous investigation on stainless steels [15], but modified to provide a 10 mm weld pool diameter on the investigated alloys.

**Table 2.** Spot-varestraint test conditions.

Parameter	
Current	150 A
Voltage	20 V
Time	~20 s
Weld spot diameter (target)	10 mm

Cracking susceptibility is determined by measuring crack features (distance, number) on the as-tested specimen surface. Cracking will most likely appear on the maximum strain regions, as indicated in Figure 1(c). The measured Maximum Crack Distance (MCD), maximum crack length (MCL), and total number of cracks (TNC) were used to compare the alloys cracking susceptibility. Figure 1(d) illustrates the difference between MCD and MCL. The MCD is measured perpendicularly from the fusion boundary to the furthest crack tip. This methodology helps quantify the temperature gradient that expands radially from the fusion line. Increased radial heat propagation and, therefore, a shallow temperature gradient promotes, if conditions permit, liquation cracking.

**Figure 2.**
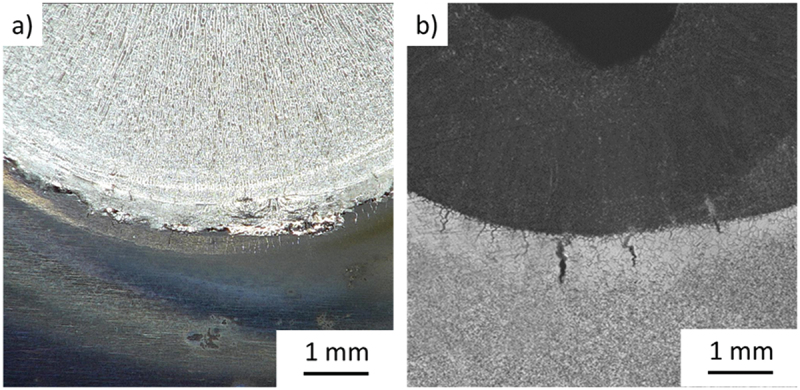
Spot Varestraint surface appearance in Alloy A; (a) As-welded, (b) Ground, polished and etched.

The cracking measurements are usually performed on the as-welded samples. However, the visualization was challenging for the investigated alloys due to excessive oxidation and slag formation despite the adequately applied gas protection, as seen in Figure 2(a). To mitigate this issue, all samples were ground and etched to better visualize the cracking features and fusion boundary, as seen in Figure 2(b).

Additional characterization was performed by optical microscopy and SEM coupled with an Energy Dispersive Spectroscopy (EDS) detector. Metallographic specimens were ground and polished up to 1um diamond paste. A 15% Nital etchant was used to reveal phases and solidification features.

Equilibrium phase diagram simulations were calculated using Calphad-based software (Thermo-Calc® 2021b) and Fe-base alloys database TCFE11. The measured chemical compositions of the alloys were used as input for the calculations. The phases used for the calculations were liquid, δ, γ, M_6_C, and k-Carbide, selected based on FeMnAl literature [2].

## 3. Results

### 3.1. Base metal characterization

**Figure 3.**
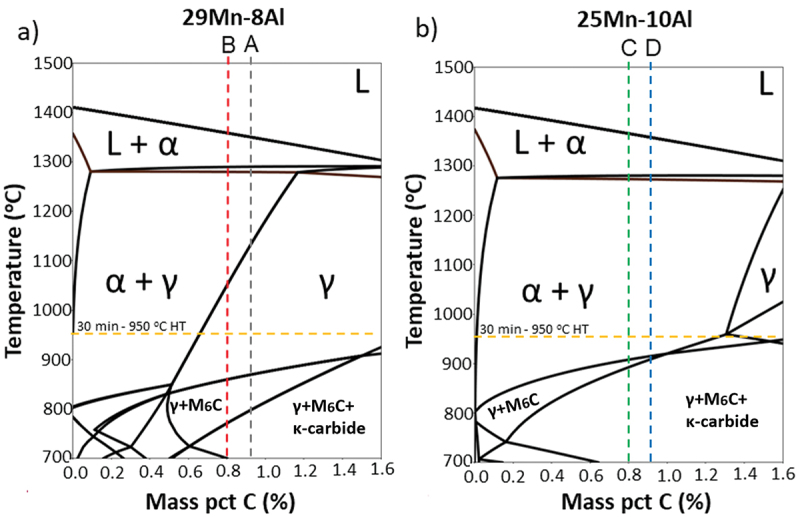
Phase diagrams for FeMnAl alloys evaluated: (a) Fe-C-29Mn-8Al diagram showing alloys a and B; (b) Fe-C-25Mn-10Al diagram showing alloys C and D.

Equilibrium phase diagrams were calculated using Thermo-Calc software to provide information regarding the phase constitution promoted by the composition differences. As shown in Figure 3, alloys A and B, due to the higher Mn and lower Al content, presented a fully austenitic (γ) microstructure after the 950°C heat treatment and quench; alloys C and D, due to the lower Mn and higher Al content presented a duplex (α-ferrite + γ-austenite) microstructure after the 950°C heat treatment and quench. Using this information, the alloys could be divided into two groups based on their base metal microstructure: Austenitic (alloys A and B), Austenite + Ferrite (alloys C and D).

**Figure 4.**
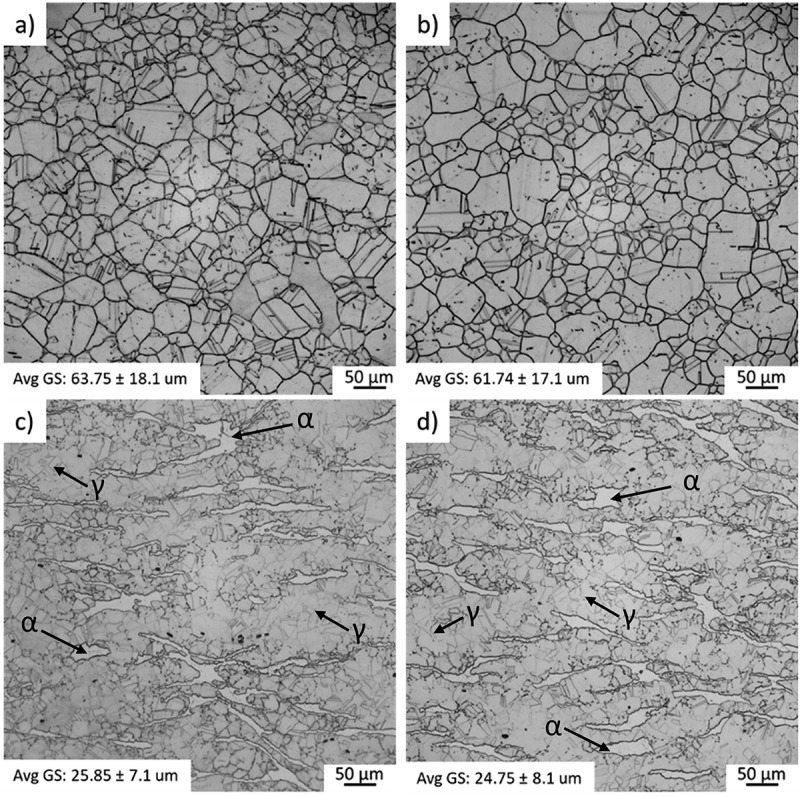
As received microstructures with respective average grain size for the alloys (a) A, (b) B, (c) C and (d) D. Optical Microscopy −15% Nital.

Micrographs of the four base metal microstructures and their respective grain size (GS) averages are shown in Figure 4. As discussed in the phase diagrams section, alloys A and B present a fully austenitic microstructure, while alloys C and D exhibit a duplex (ferrite + austenite) microstructure. It is likely that the presence of ferrite within alloys C and D promoted smaller austenitic grains compared to alloys A and B since the ferrite precipitation avoids the austenite grain growth in a competition between both phases.

**Figure 5.**
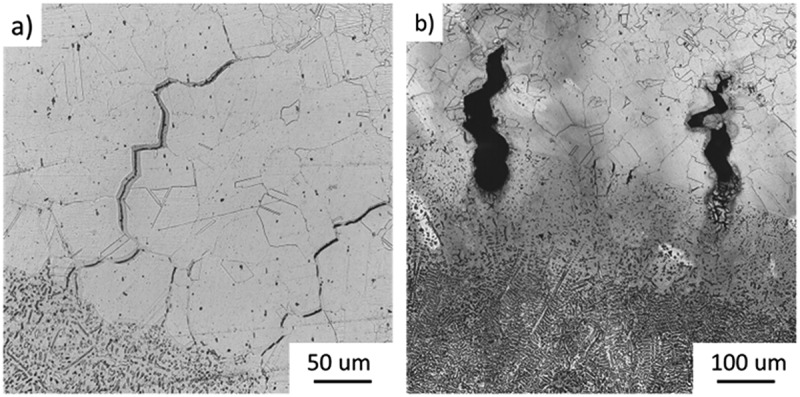
Heat affected zone liquation cracks on alloy a for an augmented strain of (a) 3%, (b) 6%. Optical Microscopy −15% Nital.

Figure 5 shows examples of cracks on as-tested samples for different strain levels. As strain levels increase, the cracking distance will also increase. However, after a specific strain level, cracks stop growing in length; instead, they get wider. Such a change in cracking behavior indicates strain saturation level has been reached. Figure 5(b) shows widened liquation cracks, suggesting that strain saturation was achieved.

**Figure 6.**
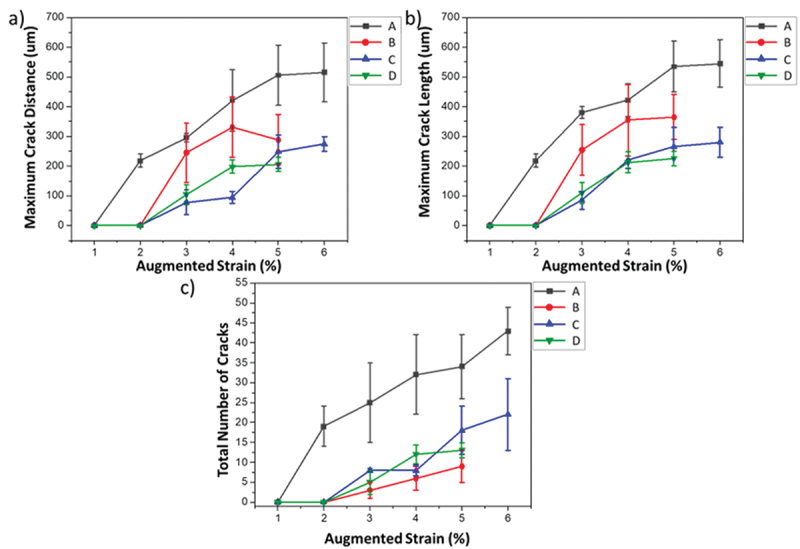
Liquation cracking metrics from the spot-varestraint test: (a) Maximum crack distance, (b) Maximum crack length and (c) Total number of cracks.

Figure 6 shows the MCD, MCL, and TNC plots for the four evaluated alloys. The vertical bars indicate the error with a 95% confidence interval. According to Figure 6(a), alloy A presented the longest cracks at its respective saturated strain region, indicating it as the most susceptible alloy of the group. Alloys C and D presented a similar behavior. Alloy C reached strain saturation at 5% and alloy D reached strain saturation at 4%, suggesting alloy C has slightly better resistance to cracking. Alloy B presented a cracking profile in between alloys A and C/D. From the interpretation of the MCL, a similar trend was observed. According to the TNC, alloy A also presented the overall greatest crack susceptibility; however, the measure could not distinguish alloys B, C, and D. As previously discussed, MCD is the most appropriate measure to compare alloys due to its physical meaning since the longer the crack propagates, the more susceptible is the alloys to the defect. The MCD indicates how far from the fusion zone the alloy is still susceptible to the liquation crack, correlating the distance and temperature.

**Figure 7.**
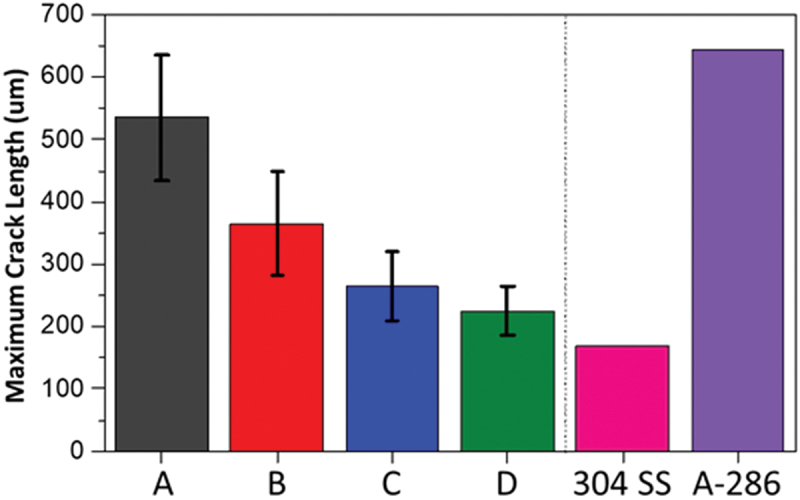
Maximum crack length at 5% augmented strain for the four FeMnAl alloys investigated. 304 SS and A-286 results from the literature [15] added for comparison purposes.

In order to better compare each alloy’s cracking behavior, the MCL at 5% strain (the strain in which all alloys reach saturation) is plotted in Figure 7. As previously discussed, alloy A was the most susceptible, followed by alloys B, C, then D. Lippold et al. [15] investigated the behavior of several austenitic and duplex stainless steels using spot-Varestraint. The MCL at 5% strain for 304 SS and A-286 determined by Lippold et al. [15] are plotted alongside the FeMnAl data in Figure 7. According to the results, alloy A presented a behavior similar to the well-known crack-susceptible A-286. Alloy D presented a behavior that was comparable to the crack-resistant 304 SS.

**Figure 8.**
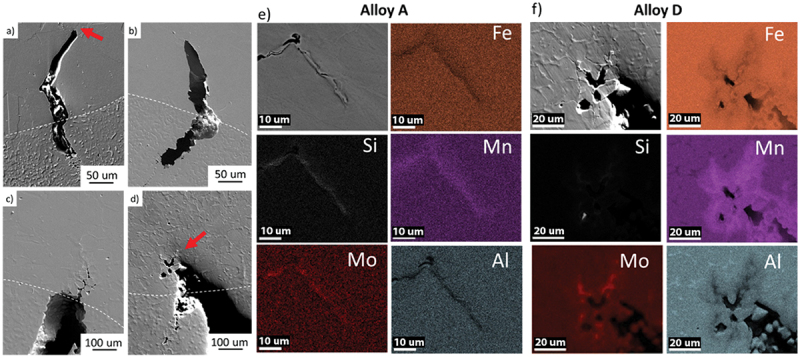
SEM images of liquation cracks within the four FeMnAl alloys: (a) alloy A, (b) alloy B, (c) alloy C and (d) alloy D. EDS composition maps of the crack tips for alloy (e) Most susceptible – alloy a and (f) Lest susceptible – alloy D.

Figure 8 shows examples of liquation cracks for the four alloys evaluated acquired using SEM. A dotted line was included to indicate the fusion boundary on each figure. Most of the cracks started within the fusion zone, suggesting susceptibility to solidification cracking as well. EDS composition maps were performed on crack tips (red arrow) of the most (A) and least (D) susceptible alloys, as seen in Figure 8. Qualitatively, both the most and least susceptible alloys presented similar elements on their crack tips: Mn, Si and Mo, and depletion of Fe and Al.

Keil et al. [19], while performing Self-Restrained Testing on a Fe-17.8Mn-1.33Al-0.59C alloy, observed high susceptibility to liquation cracking due to the presence of numerous cracks in the heat-affected zone. Using electron probe microanalysis (EPMA), the author observed high concentrations of Mn near the crack tip. The increase of Mn content was coupled with a decrease in Fe content, similar to what is observed in Figure 8.

P and S could not be accurately identified due to the small amount presented. Also, given that Lα and Kα energies of Mo and S are very close, the EDS maps do not conclusively prove the segregation of S to the grain boundaries. In general steels, S significantly influences solidification/liquation cracking by forming low-melting compounds that generate liquid films in the interdendritic regions or on grain boundaries upon reheating. However, it is suggested that S would have a negligible effect due to the high percentage of Mn, forming MnS at the expense of low-melting sulfide films. Alvarez de Toledo et al. [20] developed a critical value of Mn/S based on literature data and their own results from rolling continuous cast billets. The study showed that steel grades with a high Mn/S ratio, or more specifically higher Mn, are not as prone to hot cracking caused by S when compared to steels with a low Mn/S ratio.

**Figure 9.**
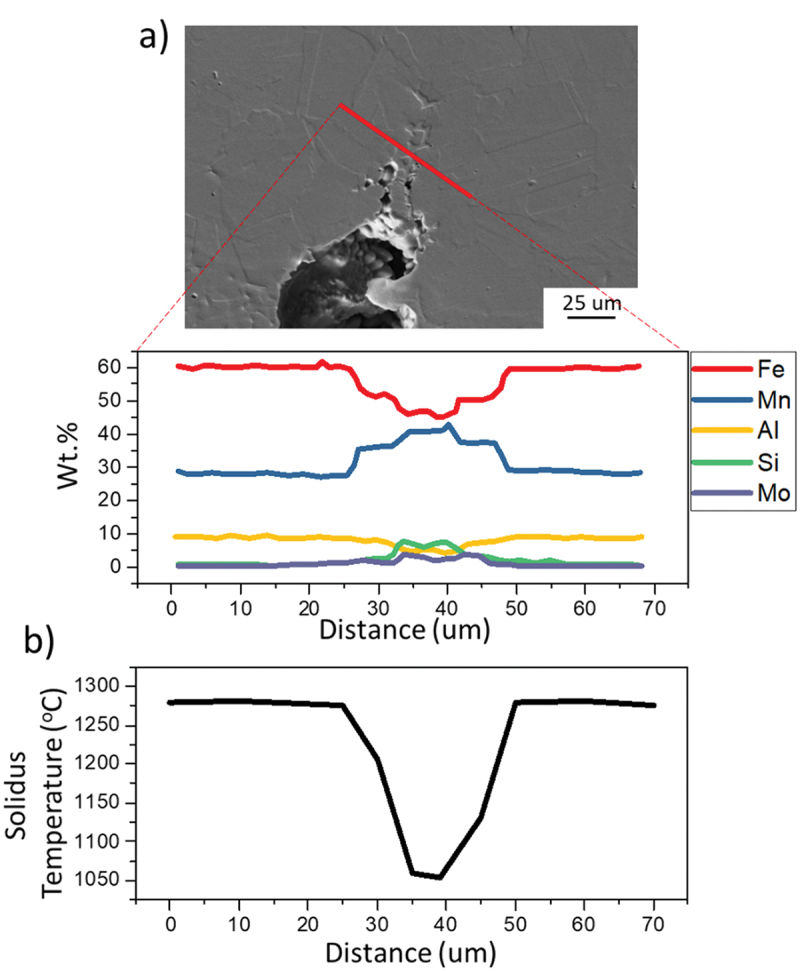
Liquation crack tip composition evaluation on alloy A. (a) EDS line scan on a crack tip in alloy a most susceptible; (b) Variation of solidus temperature as a function of distance corresponding to the EDS line scan.

A quantitative analysis was performed on the most susceptible alloy A to evaluate the effect of the observed elements on its solidus temperature. Figure 9(a) shows the EDS line region and the resulting element quantification on the liquation crack tip. As suggested by the previous maps, Fe and Al were depleted while Mn, Si, and Mo increased. Using CALPHAD, the solidus temperature for increasing Mn, Si, and Mo contents was calculated. As shown in Figure 9(b), the composition change promoted a reduction of local solidus temperatures, leading to liquation cracking formation.

**Figure 10.**
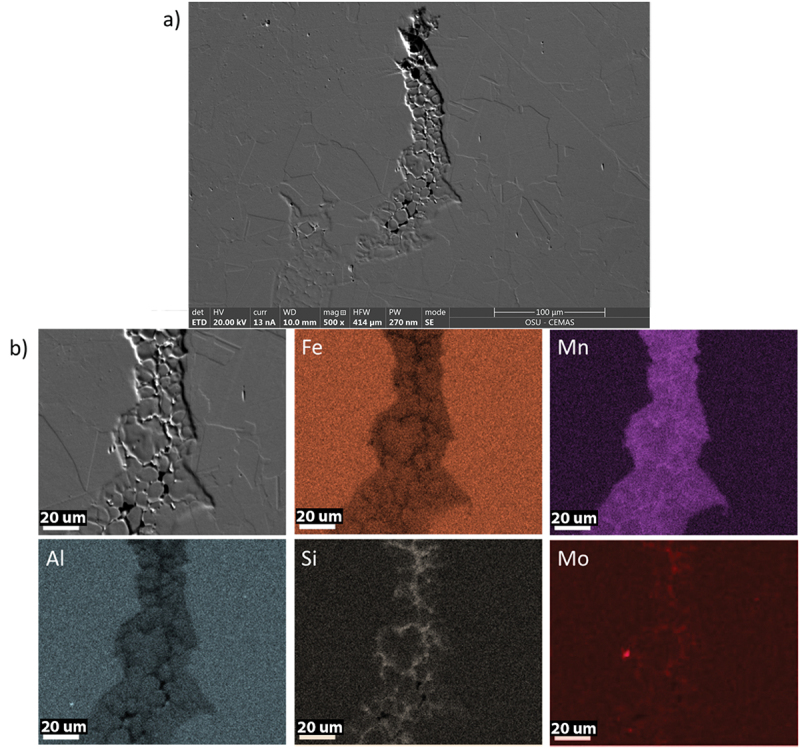
Backfilled crack analysis on alloy A. (a) Overview of the backfilled crack, (b) Composition maps revealing the distribution of Fe, Mn, Al, Si and Mo in the backfilled crack.

Some cracks were partially or fully filled by liquid metal, as seen in Figure 10. Those backfilled liquation cracks show the presence of a two-phase structure, likely a low-melting Mn-SiMo eutectic. It can be seen that there was a large volume of eutectic at the crack end. Inversely, the eutectic was nearly absent at the root of the crack. Also, the presence of the eutectic is greater on the center of the backfilled cracks due to the solidification sequence. Similar to observed in Figure 9, those regions presented a higher Mn content than the base metal. According to the phase diagram in Figure 3, it is suggested that the SiMo phase could be a SiMo-rich M_6_C carbide. The M_6_C carbide composition was calculated using equilibrium and non-equilibrium (Scheil solidification) calculations. Both conditions, as seen in Table 3, showed that M_6_C was composed of Fe-Mo-Si-C with a similar stoichiometry.

**Table 3.** Expected M_6_C composition calculated under equilibrium and non-equilibrium conditions.

M_6_C Composition (mol %)	Fe	C	Mn	Al	Si	Mo
Equilibrium	28.9	14.2	0	0	27.6	29.1
Non-Equilibrium (Scheil)	28.9	14.2	0	0	28.1	28.6

### 3.2. Liquation cracking of FeMnal

#### 3.2.1. Effect of chemical composition

The mechanisms used to describe HAZ liquation cracking can be divided into two general categories: those that support a grain boundary penetration mechanism and those in which grain boundary segregation is more significant.

The segregation model in its simplest form provides for solute/impurity element segregation to grain boundaries, thereby reducing the melting temperature of the boundary relative to the surrounding matrix. Above a critical temperature during the HAZ thermal cycle, preferential melting occurs along these boundaries, effectively embrittling a narrow region of the HAZ adjacent to the weld fusion zone. According to the EDS results, severe segregation of Mn, Mo, and Si was observed on the liquation cracks of the FeMnAl alloys explored. As demonstrated, such segregation reduces the local solidus temperature.

According to the results presented, compositions with a higher presence of Mn (A and B), an element that was extensively observed in crack regions, tended to be more susceptible to HAZ liquation cracking. This suggests that as more Mn is available within the system, more segregation is expected, resulting in increased liquation cracking susceptibility. Similarly, the alloys with higher C content, a well-known deleterious element during welding, were also more susceptible. C tends to segregate to grain boundaries and form low-melting phases, as observed by Saha et al. [21]. Si and Mo also could be contributing to the alloy’s overall susceptibility; however, their effect could not be measured in the present work.

The actual mechanism by which solute/impurity atoms segregate to the HAZ grain boundaries is unclear [15]. A possible mechanism for grain boundary segregation invokes equilibrium diffusion, where the segregated region is localized within a few atom diameters on either side of the boundary. This type of grain boundary segregation is driven by the free energy difference between a solute or impurity atom in a matrix site vs. a grain boundary site. This segregation is further enhanced when the solute/impurity element is highly surface-active and/or exhibits low solubility in the base metal. Alternatively, in the boundary-sweeping theory, as grain boundaries migrate upon heating above the threshold grain growth temperature, solute and/or impurity atoms are swept into the boundaries and are dragged along as grain growth proceeds. Therefore, surface-active and/or elements that exhibit low solubility in the matrix would have the highest probability of being swept into and assimilated with the boundary. Also, it is suggested that the mechanisms do not operate individually but rather coincidentally. More details about the segregation mechanisms can be found in Lippold’s work in HAZ Cracking of Stainless Steels [15].

#### 3.2.2. Constitutional liquation of second phases (penetration mechanism)

The penetration mechanism for HAZ liquation cracking involves the interaction of a migrating HAZ grain boundary with liquating matrix particles such as carbides, sulfides, borides, etc. The metallurgical basis for this mechanism is a phenomenon known as constitutional liquation. The κ-carbide particles, which could be potentially harmful to liquation cracking, were observed by Kim et al. [18] on FeMnAl Varestraint cracks. According to the author, tens to hundreds of nano-sized κ-carbide precipitates were created along the grain boundaries at HAZ liquation cracks. In FeMnAl alloys, the intergranular κ-carbide is usually undesirable, while the intragranular nano-sized κ-carbide is popular for improving mechanical properties [22]. However, due to the singular temperature/segregation conditions on the HAZ, some nano-sized intergranular κ-carbide could be generated.

Kim et al. [18] observed the liquation cracks associated with κ-carbide were only found 1–1.5 mm away from the fusion line. In the present work, cracking was observed starting at the fusion boundary, where temperatures around 1350°C are expected. Such temperatures are not compatible with κ-carbide formation (considering κ-carbide precipitates in the range of 450–900°C). Moreover, κ-carbide formation is enhanced by the presence of Al (a κ-carbide creation promoting element), which could potentially increase the liquation cracking susceptibility of the alloys tested. However, higher Al content alloys (C and D) presented a better cracking resistance in the present work. Although Al’s effect on κ-carbides precipitation has been disregarded, increasing Al content may operate on other properties that effect liquation cracking, such as phase constitution and element diffusivity. As observed in Figure 3, the lower presence of Al in alloys A and B promotes a fully-γ microstructure on temperatures below ~1100°C, which could contribute to the longer cracks observed on those alloys because a γ-structure is more susceptible to HAZ cracks than a fully ferritic (α) or duplex (α + γ) structure [15]. Additionally, Al was reported to alter the behavior of C within Mn-steels, as observed by Zuidema et al. The author stated that Al increases the metastable solubility of C in austenite by reducing carbon activity and diffusivity. Such behavior could reduce the concentration C on grain boundaries, reducing its negative effect on weldability.

#### 3.2.3. Grain size/microstructure

Grain size is a critical factor in liquation cracking susceptibility. The larger the average grain size of the base metal, the more precipitates at the grain boundaries are expected, resulting in more serious liquation [14,15,23]. Two major effects are related to this phenomenon. First, Woo et al. [24], studying the effect of grain size on heat-affected zone cracking susceptibility, observed improvement through grain refinement. Basically, the grain boundary liquation is reduced through the proportional reduction of Laves clusters at the grain boundaries. Secondly, as observed by Miyazaki et al. [25], when the grain size becomes larger, the solidus temperature at the crack tip gradually decreases, and cracks were observed even at temperatures much lower than the solidus temperature of the base metal. The average grain size of the alloys C and D material is finer than A and B, contributing to a better liquation cracking resistance.

**Figure 11.**
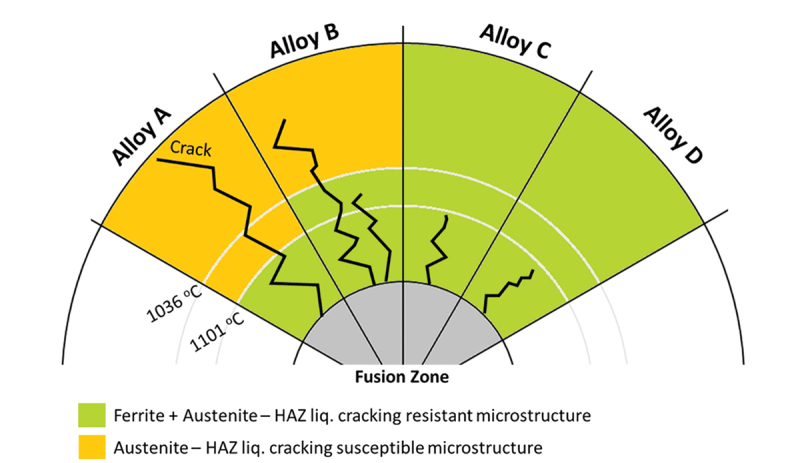
Liquation cracking schematics based on the respective phase transformation temperatures of alloys A, B, C, and D.

Regarding the microstructure, Kujanpaa et al. [26] noted the relationship between weld ferrite content and HAZ liquation cracking. The rationale was that ferrite along HAZ grain boundaries inhibits wetting by liquid films and limits diffusion of impurity elements. Another critical effect of ferrite formation along austenite grain boundaries is the restriction of grain growth. Figure 11 illustrates the effect of the microstructure on the crack distance observed experimentally. Based on the phase diagram calculation, isothermal lines show the phase transformation location for each alloy, related to the calculated temperatures in Figure 3. All alloys present a duplex microstructure close to the fusion zone; however, differences are observed as the temperature gradient proceeds. Alloy A presents an early transformation at 1101°C, providing a more susceptible microstructure sooner than other alloys, facilitating cracking propagation. Alloys C and D are expected to resist liquation cracking due to the constant ferrite presence as temperatures decrease. Due to the later transformation into a fully austenitic matrix, Alloy B presented an in-between behavior, which also agrees with the presented results.

## 4. Conclusions

In this study, an investigation of cracking in the HAZ of FeMnAl steel was conducted using spot-Varestraint testing, with a particular focus on the effect of major alloying element (C, Mn and Al) variations. The following conclusions were drawn:

- The alloy with the higher Mn, C, and lower Al (alloy A) presented the longest cracks in the saturated strain region, indicating it as the most susceptible alloy in the investigated group. Conversely, the alloy with lower Mn, C, and Al (alloy D) presented the most resistant behavior.
- Element quantification from an EDS line scan of a liquation crack tip showed the depletion of Fe and Al and increased concentrations of Mn, Si, and Mo. Additions of Mn, Si, and Mo promote a reduction of local solidus temperature, which contributes to increased liquation cracking susceptibility.
- Lower Al content within FeMnAl alloys promotes a fully-γ microstructure at low temperatures, which corresponds to the longer cracks as a γ-matrix is more susceptible to HAZ cracking than a fully ferritic (α) or duplex (α + γ) microstructure.
- The observed conditions that produce a more HAZ liquation cracking resistant FeMnAl alloy are as follows: the combination of lower Mn and higher Al contents to provide a duplex microstructure and reduced grain size.
